# Analysis of *Plasmodium falciparum* Na^+^/H^+^ exchanger (*pfnhe1*) polymorphisms among imported African malaria parasites isolated in Wuhan, Central China

**DOI:** 10.1186/s12879-019-3921-7

**Published:** 2019-04-29

**Authors:** Kai Wu, Yi Yao, Fang Chen, Mingxing Xu, Guangquan Lu, Tingting Jiang, Ziyu Liu, Weixing Du, Fang Li, Rugui Li, Huabing Tan, Jian Li

**Affiliations:** 1 Department of Schistosomiasis and Endemic Diseases, Wuhan City Center for Disease Prevention and Control, Wuhan, 430015 People’s Republic of China; 2 Department of Infectious Diseases, Renmin Hospital; Department of Human Parasitology, School of Basic Medical Sciences, Hubei University of Medicine, Shiyan, 442000 People’s Republic of China; 3 Department of Pediatrics, Shiyan Hospital of Traditional Chinese, Shiyan, 442000 People’s Republic of China

**Keywords:** *Plasmodium falciparum*, Na^+^/H^+^ exchanger, Quinine resistance, Microsatellite

## Abstract

**Background:**

Quinine (QN) remains an effective drug for malaria treatment. However, quinine resistance (QNR) in *Plasmodium falciparum* has been reported in many malaria-endemic regions particularly in African countries. Genetic polymorphism of the *P. falciparum* Na^+^/H^+^ exchanger (*pfnhe1*) is considered to influence QN susceptibility. Here, ms4760 alleles of *pfnhe1* were analysed from imported African *P. falciparum* parasites isolated from returning travellers in Wuhan, Central China.

**Methods:**

A total of 204 dried-blood spots were collected during 2011–2016. The polymorphisms of the *pfnhe1* gene were determined using nested PCR with DNA sequencing.

**Results:**

Sequences were generated for 99.51% (203/204) of the PCR products and 68.63% (140/204) of the isolates were analysed successfully for the *pfnhe1* ms4760 haplotypes. In total, 28 distinct ms4760 alleles containing 0 to 5 DNNND and 1 to 3 NHNDNHNNDDD repeats were identified. For the alleles, ms4760–1 (22.86%, 32/140), ms4760–3 (17.86%, 25/140), and ms4760–7 (10.71%, 15/140) were the most prevalent profiles. Furthermore, 5 undescribed ms4760 alleles were reported.

**Conclusions:**

The study offers an initial comprehensive analysis of *pfnhe1* ms4760 polymorphisms from imported *P. falciparum* isolates in Wuhan. *Pfnhe1* may constitute a good genetic marker to evaluate the prevalence of QNR in malaria-endemic and non-endemic regions.

## Background

Quinine (QN), a natural quinoline derivative from Cinchona bark, has been widely used in malaria-endemic regions for several centuries to treat severe malaria cases or malaria in the first trimester of pregnancy [1]. Following the guidelines of the World Health Organization (WHO), many malaria-endemic countries and regions have adopted artemisinin based combination therapies (ACTs) as first-line treatments since 2001 [2]. QN has been used as a second-line drug combined with doxycycline, tetracycline or clindamycin for uncomplicated malaria [3, 4]. In addition, with failures of previous therapies and limited availability of ACTs, QN is increasingly used as a first-line drug for the treatment of uncomplicated malaria in Uganda [5]. Although QN was an effective antimalaria drug, it has gradually decreased in sensitivity for malaria treatment [6–10]. In the early 1960s, the first QN clinical failure cases were reported in Brazil and Asia [11, 12]. Since then, more and more failure cases have been reported in Southeast Asia [6, 7], South America [6], and Africa [8–10].

To date, some genes associated with drug resistance have been identified, including *Plasmodium falciparum* chloroquine resistance transporter (*pfcrt*) [13], multidrug resistance 1 (*pfmdr1*) [14], multidrug resistance associated protein (*pfmrp1*) [15], dihydrofolate reductase (*pfdhfr*) [16], dihydropteroate synthase (*pfdhps*) [17], sodium/hydrogen exchanger (*pfnhe1*) [18], and kelch protein 13 (*pfK13)* [19]. However, the phenotype of quinine resistance (QNR) is complex and appears to be influenced by multiple genes located at different loci [20, 21]. Although the molecular mechanisms of QNR remain unclear, QN susceptibility in vitro is linked to polymorphisms in multiple genes, including *pfmdr1* [14], *pfcrt* [22, 23], *pfmrp* [24] and *pfnhe1* [20]. QNR-associated single nucleotide polymorphisms (SNPs) have been identified in *pfmdr1* [14] at codons 86, 184, 1042, and 1246 and in *pfcrt* [23] at codon 76. Moreover, in our previous study, only 33.68% (65/193) of the isolates carried the N_86_Y_184_ wild-type allele in *pfmdr1* and 49.43% (87/176) of the isolates carried the *pfcrt* K76 T mutation, suggesting moderate sensitivity to QN in Africa [25]. However, evidence for the involvement of *pfnhe1* in QNR is still limited. A previous study demonstrated that *pfnhe1* might be involved in QNR [26]. For the *pfnhe1* gene, SNPs at codons 790, 894 and 950 and microsatellite variations in three different repeat sequences (msR1, ms3580 and ms4760) located on chromosome 13 of the *P. falciparum* genome have been identified [4]. However, these SNPs and microsatellite variations in msR1 and ms3580 showed no significant association with QN susceptibility [4]. Ms4760 with two or more DNNND repeats (Block II) was associated with a higher QN inhibition [20, 27]. Another study illustrated that the increased number of DNNND repeats (Block II) was related to a decreasing trend in QN susceptibility, and the increased number of NHNDNHNNDDD repeats (Block V) was related to increased QN susceptibility [28].

In light of these reports, we investigated the prevalence of *pfnhe1* in patients who were infected with *P. falciparum* returning from different African countries and assessed whether *pfnhe1* can be used as a molecular marker of QNR.

## Methods

### Clinical sample collection and genomic DNA extraction

In total, 204 dried-blood spots (DBSs) were collected from patients who were infected with *P. falciparum* returning from Africa and confirmed by the Wuhan Centers for Disease Prevention and Control (CDC) during 2011–2016. These samples were examined using One Step Malaria HRP2/pLDH (P.f/Pan) tests (Wondfo, Guangzhou, China) and Giemsa-stained thick and thin peripheral blood smear examinations. The identities of Plasmodium spp. were confirmed by real-time fluorescent quantitative PCR. Genomic DNA (gDNA) of uncomplicated *P. falciparum* isolates was extracted from DBSs by using a TIANamp Blood DNA Kit (Tiangen Biotech Co., Ltd., Beijing, China) according to the manufacturer’s instructions. To characterize the microsatellite repeats in *pfnhe1*, the ms4760 region (482 bp) of the *pfnhe1* gene was amplified by nested PCR in 204 samples.

### Nested PCR amplification of the *pfnhe1* gene

DNA was amplified by nested PCR (Bio-Rad Mini MJ thermal cycler). The primary primers including NHE-A and NHE-B, and the secondary primers containing NHE-C and NHE-D were described in a previous study [29–31]. No negative control was set for the PCR. For the primary round of PCR, 0.5 μl of DNA was amplified with 10 μl 2× NovoStar Green PCR Mix (1.25 U/25 μl NovoStar Taq DNA Polymerase, 0.4 mM dNTP mixture, 2 × PCR buffer, and 4 mM Mg^2+^), 0.5 μl forward primer (10 μM), 0.5 μl reverse primer (10 μM), and sterile ultrapure water to a final volume of 20 μl. The gene target was amplified under the following conditions for the first run: initial denaturation at 95 °C for 3 min; followed by 30 cycles of 95 °C for 30 s, 55 °C for 30 s, and 72 °C for 1 min; and a final extension at 72 °C for 5 min. For the second round of PCR, 1.0 μl primary PCR products were amplified with a 50 μl reaction system, including 25 μl 2× NovoStar Green PCR Mix, 1.0 μl forward primer (10 μM), 1.0 μl reverse primer (10 μM), and 22 μl H_2_O. Secondary run conditions were as follows: initial denaturation at 95 °C for 3 min; followed by 30 cycles of 95 °C for 30 s, 55 °C for 30 s, and 72 °C for 30 s; and a final extension at 72 °C for 5 min.

Five microliters (5 μl) of nested PCR products were resolved by electrophoresis in 1% agarose gels, and examined for quality under a UV light. Samples with bright bands were selected for DNA sequencing (Genewiz, Soochow, China). Sequences were then translated using the Edit Sequence tool and aligned using the MEGALIGN programme with DNAstar (DNASTAR Inc., Madison, WI, USA) software. The amino acid sequences were compared with the reference sequence from PlasmoDB (https://plasmodb.org/plasmo/) under Gene ID.: PF3D7_1303500.

### Data analysis

All statistical data were analysed using SPSS 18 (SPSS Inc., Chicago, IL, USA) and GraphPad Prism (version 5.01). *Pfnhe1* ms4760 profiles were analysed, including the numbers of Block II and Block V. The prevalence of haplotypes between years and areas were compared using Pearson’s chi-square tests or Fisher’s exact test when applicable [32]. Specifically, Pearson’s chi-square tests were Yates corrected and Fisher’s exact tests were one-tailed. A *P* value less than 0.05 was considered significant.

## Results

### General information

**Fig. 1:**
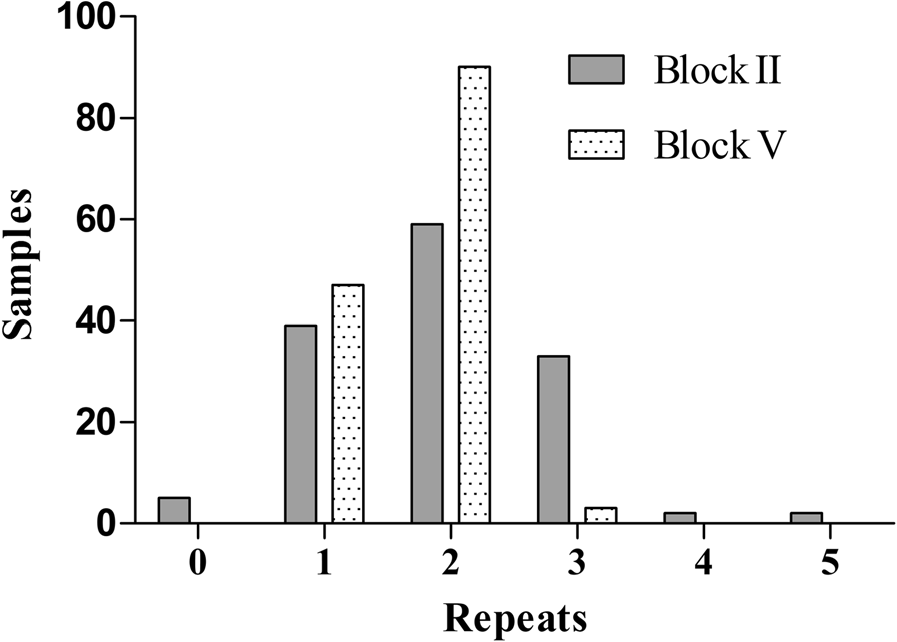
The distribution of *pfnhe1* profiles according to the number of repeats in Block II (DNNND) and Block V (NHNDNHNNDDD)

DNA blood samples from 204 patients with uncomplicated *P. falciparum* malaria were collected and tested. All 204 samples were successfully amplified by nested PCR. Sequences were generated for 99.51% (203/204) of the PCR products and included 63 poor-quality sequencing products. Finally, 68.63% (140/204, 95% CI: 78.77 to 88.87%) of PCR products were analysed for *pfnhe1* ms4760 haplotypes. In the 140 studied samples, the number of DNNND repeats (Block II) ranged from zero to five, with one, two and three repeats being more common and accounting for 27.86% (39/140), 42.14% (59/140) and 23.57% (33/140) of these samples, respectively (Fig. 1); combined repeats in Block II accounted for 93.57% (131/140) of the samples. The number of NHNDNHNNDDD repeats (Block V) ranged from one to three, with one repeat accounting for 33.57% (47/140) of the samples and two repeats accounting for 64.29% (90/140) of the samples; NHNDNHNNDDD represented the dominant repeat in these isolates (Fig. 1). Combined one and two repeats in Block V made up 97.86% (137/140) of the samples. The number of Block II and V repeats by different regions and years are presented in Table 1 and Table 2, respectively.

**Table 1:** The observed repeats of Block II (DNNND) and Block V (NHNDNHNNDDD) from different areas of Africa

Region	Total	No. of DNNND repeats						No. of NHNDNHNNDDD repeats		
		0	1	2	3	4	5	1	2	3
West Africa	62	2 (3.23%)	14 (22.58%)	27 (43.55%)	16 (25.81%)	1 (1.61%)	2 (3.23%)	17 (27.42%)	44 (70.97%)	1 (1.61%)
Central Africa	32	0 (0.00%)	10 (31.25%)	15 (46.88%)	6 (18.75%)	1 (3.13%)	0 (0.00%)	12 (37.50%)	20 (62.50%)	0 (0.00%)
South Africa	29	2 (6.90%)	9 (31.03%)	10 (34.48%)	8 (27.59%)	0 (0.00%)	0 (0.00%)	13 (44.83%)	14 (48.28%)	2 (6.90%)
East Africa	14	1 (7.14%)	6 (42.86%)	4 (28.57%)	3 (21.43%)	0 (0.00%)	0 (0.00%)	4 (28.57%)	10 (71.43%)	0 (0.00%)
North Africa	3	0 (0.00%)	0 (0.00%)	3 (100.00%)	0 (0.00%)	0 (0.00%)	0 (0.00%)	1 (33.33%)	2 (66.67%)	0 (0.00%)
Total	140	5 (3.57%)	39 (27.86%)	59 (42.14%)	33 (23.57%)	2 (1.43%)	2 (1.43%)	47 (33.57%)	90 (64.29%)	3 (2.14%)

Note: No. stands for number

**Table 2:** The observed repeats of Block II (DNNND) and Block V (NHNDNHNNDDD) during 2011-2016

Year	Total	No. of DNNND repeats						No. of NHNDNHNNDDD repeats		
		0	1	2	3	4	5	1	2	3
2011	3	0 (0.00%)	0 (0.00%)	2 (66.67%)	1 (33.33%)	0 (0.00%)	0 (0.00%)	0 (0.00%)	3 (100.00%)	0 (0.00%)
2012	21	2 (9.52%)	2 (9.52%)	10 (47.62%)	7 (33.33%)	0 (0.00%)	0 (0.00%)	8 (38.10%)	13 (61.90%)	0 (0.00%)
2013	34	1 (2.94%)	10 (29.41%)	14 (41.18%)	9 (26.47%)	0 (0.00%)	0 (0.00%)	11 (32.35%)	22 (64.71%)	1 (2.94%)
2014	28	0 (0.00%)	10 (35.71%)	13 (46.43%)	4 (14.29%)	0 (0.00%)	1 (3.57%)	7 (25.00%)	20 (71.43%)	1 (3.57%)
2015	24	2 (8.33%)	7 (29.17%)	5 (20.83%)	7 (29.17%)	2 (8.33%)	1 (4.17%)	11 (45.83%)	13 (54.17%)	0 (0.00%)
2016	30	0 (0.00%)	10 (33.33%)	15 (50.00%)	5 (16.67%)	0 (0.00%)	0 (0.00%)	10 (33.33%)	19 (63.33%)	1 (3.33%)
Total	140	5 (3.57%)	39 (27.86%)	59 (42.14%)	33 (23.57%)	2 (1.43%)	2 (1.43%)	47 (33.57%)	90 (64.29%)	3 (2.14%)

Note: No. stands for number

### Genotyping of *pfnhe1* ms4760 microsatellite polymorphisms

**Fig. 2:**
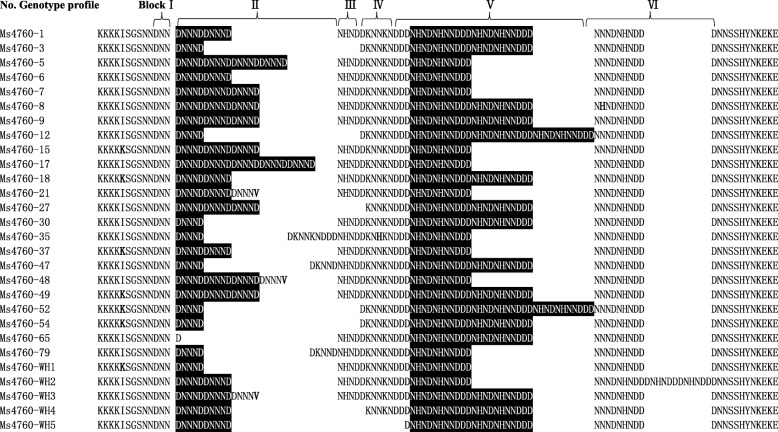
Multiple amino acid sequence alignments of 28 *pfnhe1* ms4760 genotypes. Two types of repeats, Block II (DNNND) and Block V (NHNDNHNNDDD), are highlighted. Bold letters indicate mutated amino acids

Among the 140 *P. falciparum* clinical isolates, 28 different alleles for *pfnhe1* ms4760 were observed, including five profiles not previously described (Table 3). Multiple amino acid sequence alignments of these genotypes are displayed in Fig. 2. A total of 82.14% (23/28) of the alleles contained previously described ms4760 haplotypes. The three most prevalent profiles made up 51.43% (72/140) of the isolates, including 22.86% (32/140) ms4760–1, 17.86% (25/140) ms4760–3 and 10.71% (15/140) ms4760–7. The least common genetic polymorphisms were ms4760–52, ms4760–27, ms4760–30, ms4760–47, ms4760–48, and ms4760–79, each found in 0.71% (1/140) of the isolates. The 5 previously undescribed ms4760 haplotypes were named ms4760-WH1, ms4760-WH2, ms4760-WH3, ms4760-WH4 and ms4760-WH5 (Table 3). Of these five newly observed alleles, ms4760-WH1 was the most common, accounting for 2.86% (4/140), and the other four were all found at 0.71% (1/140).

**Table 3:** Distribution of the *pfnhe1* ms4760 alleles among the Africa-imported *Plasmodium falciparum* isolates

Allele	Haplotypes	Repeated No. of		Region				
		DNNND	NHNDNHNNDDD	West Africa	Central Africa	South Africa	East Africa	North Africa
ms4760-1	32	2	2	13 (40.63%)	8 (25.00%)	6 (18.75%)	3 (9.38%)	2 (6.25%)
ms4760-3	25	1	2	9 (36.00%)	7 (28.00%)	4 (16.00%)	5 (20.00%)	0 (0.00%)
ms4760-7	15	3	1	6 (40.00%)	2 (13.33%)	5 (33.33%)	2 (13.33%)	0 (0.00%)
ms4760-6	8	2	1	3 (37.50%)	3 (37.50%)	1 (12.50%)	1 (12.50%)	0 (0.00%)
ms4760-9	7	3	2	4 (57.14%)	2 (28.57%)	1 (14.29%)	0 (0.00%)	0 (0.00%)
ms4760-18	6	2	2	6 (100.00%)	0 (0.00%)	0 (0.00%)	0 (0.00%)	0 (0.00%)
ms4760-65	5	0	2	2 (40.00%)	0 (0.00%)	2 (40.00%)	1 (20.00%)	0 (0.00%)
ms4760-54	4	1	2	2 (50.00%)	1 (25.00%)	1 (25.00%)	0 (0.00%)	0 (0.00%)
ms4760-15	4	3	1	2 (50.00%)	1 (25.00%)	1 (25.00%)	0 (0.00%)	0 (0.00%)
ms4760-37	4	2	1	1 (25.00%)	2 (50.00%)	0 (0.00%)	0 (0.00%)	1 (25.00%)
ms4760-35	3	1	1	0 (0.00%)	1 (33.33%)	2 (66.67%)	0 (0.00%)	0 (0.00%)
ms4760-49	3	3	2	3 (100.00%)	0 (0.00%)	0 (0.00%)	0 (0.00%)	0 (0.00%)
ms4760-8	2	3	2	0 (0.00%)	1 (50.00%)	0 (0.00%)	1 (50.00%)	0 (0.00%)
ms4760-12	2	1	3	1 (50.00%)	0 (0.00%)	1 (50.00%)	0 (0.00%)	0 (0.00%)
ms4760-5	2	4	1	1 (50.00%)	1 (50.00%)	0 (0.00%)	0 (0.00%)	0 (0.00%)
ms4760-17	2	5	1	2 (100.00%)	0 (0.00%)	0 (0.00%)	0 (0.00%)	0 (0.00%)
ms4760-21	2	2	1	0 (0.00%)	0 (0.00%)	2 (100.00%)	0 (0.00%)	0 (0.00%)
ms4760-52	1	1	3	0 (0.00%)	0 (0.00%)	1 (100.00%)	0 (0.00%)	0 (0.00%)
ms4760-27	1	3	2	1 (100.00%)	0 (0.00%)	0 (0.00%)	0 (0.00%)	0 (0.00%)
ms4760-30	1	1	2	1 (100.00%)	0 (0.00%)	0 (0.00%)	0 (0.00%)	0 (0.00%)
ms4760-47	1	1	2	1 (100.00%)	0 (0.00%)	0 (0.00%)	0 (0.00%)	0 (0.00%)
ms4760-48	1	3	1	0 (0.00%)	0 (0.00%)	1 (100.00%)	0 (0.00%)	0 (0.00%)
ms4760-79	1	1	1	0 (0.00%)	1 (100.00%)	0 (0.00%)	0 (0.00%)	0 (0.00%)
ms4760-WH1	4	2	1	2 (50.00%)	1 (25.00%)	1 (25.00%)	0 (0.00%)	0 (0.00%)
ms4760-WH2	1	1	1	0 (0.00%)	0 (0.00%)	0 (0.00%)	1 (100.00%)	0 (0.00%)
ms4760-WH3	1	2	2	0 (0.00%)	1 (100.00%)	0 (0.00%)	0 (0.00%)	0 (0.00%)
ms4760-WH4	1	2	2	1 (100.00%)	0 (0.00%)	0 (0.00%)	0 (0.00%)	0 (0.00%)
ms4760-WH5	1	2	2	1 (100.00%)	0 (0.00%)	0 (0.00%)	0 (0.00%)	0 (0.00%)
Total	140			62 (44.29%)	32 (22.86%)	29 (20.71%)	14 (10.00%)	3 (2.14%)

Note: No. stands for number

### Geographical distribution of ms4760 microsatellite polymorphisms

The distribution included twenty distinct ms4760 alleles from West Africa, 14 alleles from South Africa, 14 alleles from Central Africa, and 7 alleles from East Africa (Tables 3 and 4). As the most common allele, ms4760–1 was present in all regions of Africa but not in all countries (Table 3). Conversely, several alleles were only seen in partial regions or a specific country (Table 3 and 4). Only the three most numerous alleles (ms4760–1, ms4760–3, ms4760–7) were analysed using Pearson’s chi-square test. However, the three alleles were not significantly associated with four regions in Africa and excluded North Africa (*P* > 0.05). The ms4760 alleles in the parasite isolates from Nigeria, Congo, Angola, and Liberia accounted for 47.86% (67/140) of the samples. Furthermore, a combination of samples from Nigeria (50%, 14/28), Congo (32.14%, 9/28), Liberia (25%, 7/28), and Angola (21.43%, 6/28) was responsible for 67.86% (19/28) of the alleles. Although only 8 samples were included in the analysis for Mozambique, various ms4760 alleles (28.57%, 8/28) were detected. The same phenomenon can be observed for Guinea, Ghana, Zambia, Cameroon and Uganda (Table 4).

**Table 4:** Distribution of pfnhe1 ms4760 alleles from different areas of Africa

Region	Country	Total	Allele
West Africa	Nigeria	21	ms4760-1, 3, 5, 6, 7, 9, 15, 17, 18, 37, 47, 54, 65, WH4
	Liberia	14	ms4760-1, 3, 6, 7, 15, 27, WH5
	Guinea	7	ms4760-1, 3, 17, 18, 54, 65
	Sierra Leone	6	ms4760-1, 7, 18, 49
	Ghana	6	ms4760-3, 12, 18, 30, 49, WH2
	Ivory Coast	3	ms4760-3, 9, 49
	Benin	3	ms4760-1, 18, WH2
	Niger	2	ms4760-1, 7
	Subtotal	62	ms4760-1, 3, 5, 6, 7, 9, 12, 15, 17, 18, 27, 30, 37, 47, 49, 54, 65, WH2, WH4, WH5
South Africa	Angola	15	ms4760-1, 3, 6, 7, 35, 65
	Zambia	6	ms4760-1, 3, 9, 21, 35
	Mozambique	8	ms4760-3, 12, 15, 21, 48, 52, 54, WH2
	Subtotal	29	ms4760-1, 3, 6, 7, 9, 12, 15, 21, 35, 48, 52, 54, 65, WH2
Central Africa	Congo	17	ms4760-1, 3, 5, 6, 9, 37, 54, 79, WH2
	Equatorial Guinea	7	ms4760-1, 3, 6, 8, WH3
	Cameroon	6	ms4760-1, 3, 7, 9, 35
	Gabon	2	ms4760-3, 15
	Subtotal	32	ms4760-1, 3, 5, 6, 7, 8, 9, 15, 35, 37, 54, 79, WH2, WH3
East Africa	Uganda	6	ms4760-1, 3, 6, 8, 65
	South Sudan	2	ms4760-1, 3
	Tanzania	3	ms4760-3, 7
	Ethiopia	2	ms4760-3, WH1
	Rwanda	1	ms4760-3
	Subtotal	14	ms4760-1, 3, 6, 7, 8, 65, WH1
North Africa	Sudan	2	ms4760-1, 37
	Libya	1	ms4760-1
	Subtotal	3	ms4760-1, 37

### Annual distribution of ms4760 microsatellite polymorphisms

A total of 28 different ms4760 alleles were observed among the 140 imported African *P. falciparum* parasites isolated in Wuhan, China, between 2011 and 2016. These included two distinct ms4760 alleles in 2011, 13 alleles in 2012, 17 alleles in 2013, 12 alleles in 2014, 11 alleles in 2015, and 12 alleles in 2016 (Table 5). There was the highest number of alleles in 2013. Specifically, ms4760-WH1 was seen in 2013, 2014 and 2016. However, ms4760-WH2, WH3, WH4, and WH5 were only found in a single year. Of note, ms4760–1 was the most prevalent allele (23%; 32/140), and it was the only allele that was consistently observed throughout the 6-year study period, followed by ms4760–3 (18%; 25/140), and ms4760–7 (11%; 15/140; Table 5). These three most common alleles were further analyzed using Pearson’s chi-square test or Fisher’s exact test. There was a statistically significant difference in the prevalence of ms4760–1 between 2011 and 2015 (*P* = 0.049). No significant associations were seen among years for the other alleles (*P* > 0.05). A significant association between ms4760–1 and ms4760–7 was also observed in 2015 (*X*^*2*^ = 6.020, *P* = 0.014). No significant associations were seen among the alleles for other years (*P* > 0.05).

**Table 5:** Distribution of *pfnhe1* ms4760 alleles during 2011-2016

Allele	Total	No. of haplotypes (%)					
		2011	2012	2013	2014	2015	2016
ms4760-1	32	2 (6.25%)	3 (9.38%)	8 (25.00%)	9 (28.13%)	2 (6.25%)	8 (25.00%)
ms4760-3	25	0 (0.00%)	1 (4.00%)	6 (24.00%)	6 (24.00%)	5 (20.00%)	7 (28.00%)
ms4760-7	15	0 (0.00%)	3 (20.00%)	3 (20.00%)	1 (6.67%)	6 (40.00%)	2 (13.33%)
ms4760-6	8	0 (0.00%)	4 (50.00%)	2 (25.00%)	0 (0.00%)	0 (0.00%)	2 (25.00%)
ms4760-9	7	1 (14.29%)	1 (14.29%)	0 (0.00%)	2 (28.57%)	1 (14.29%)	2 (28.57%)
ms4760-18	6	0 (0.00%)	1 (16.67%)	1 (16.67%)	1 (16.67%)	2 (33.33%)	1 (16.67%)
ms4760-65	5	0 (0.00%)	2 (40.00%)	1 (20.00%)	0 (0.00%)	2 (40.00%)	0 (0.00%)
ms4760-54	4	0 (0.00%)	1 (25.00%)	0 (0.00%)	2 (50.00%)	1 (25.00%)	0 (0.00%)
ms4760-15	4	0 (0.00%)	1 (25.00%)	1 (25.00%)	1 (25.00%)	0 (0.00%)	1 (25.00%)
ms4760-37	4	0 (0.00%)	0 (0.00%)	0 (0.00%)	0 (0.00%)	1 (25.00%)	3 (75.00%)
ms4760-35	3	0 (0.00%)	0 (0.00%)	1 (33.33%)	1 (33.33%)	1 (33.33%)	0 (0.00%)
ms4760-49	3	0 (0.00%)	0 (0.00%)	3 (100.00%)	0 (0.00%)	0 (0.00%)	0 (0.00%)
ms4760-8	2	0 (0.00%)	1 (50.00%)	1 (50.00%)	0 (0.00%)	0 (0.00%)	0 (0.00%)
ms4760-12	2	0 (0.00%)	0 (0.00%)	0 (0.00%)	1 (50.00%)	0 (0.00%)	1 (50.00%)
ms4760-5	2	0 (0.00%)	0 (0.00%)	0 (0.00%)	0 (0.00%)	2 (100.00%)	0 (0.00%)
ms4760-17	2	0 (0.00%)	0 (0.00%)	0 (0.00%)	1 (50.00%)	1 (50.00%)	0 (0.00%)
ms4760-21	2	0 (0.00%)	0 (0.00%)	1 (50.00%)	1 (50.00%)	0 (0.00%)	0 (0.00%)
ms4760-52	1	0 (0.00%)	0 (0.00%)	1 (100.00%)	0 (0.00%)	0 (0.00%)	0 (0.00%)
ms4760-27	1	0 (0.00%)	1 (100.00%)	0 (0.00%)	0 (0.00%)	0 (0.00%)	0 (0.00%)
ms4760-30	1	0 (0.00%)	0 (0.00%)	1 (100.00%)	0 (0.00%)	0 (0.00%)	0 (0.00%)
ms4760-47	1	0 (0.00%)	0 (0.00%)	0 (0.00%)	0 (0.00%)	0 (0.00%)	1 (100.00%)
ms4760-48	1	0 (0.00%)	0 (0.00%)	1 (100.00%)	0 (0.00%)	0 (0.00%)	0 (0.00%)
ms4760-79	1	0 (0.00%)	0 (0.00%)	0 (0.00%)	0 (0.00%)	0 (0.00%)	1 (100.00%)
ms4760-WH1	4	0 (0.00%)	0 (0.00%)	1 (25.00%)	2 (50.00%)	0 (0.00%)	1 (25.00%)
ms4760-WH2	1	0 (0.00%)	0 (0.00%)	1 (100.00%)	0 (0.00%)	0 (0.00%)	0 (0.00%)
ms4760-WH3	1	0 (0.00%)	1 (100.00%)	0 (0.00%)	0 (0.00%)	0 (0.00%)	0 (0.00%)
ms4760-WH4	1	0 (0.00%)	0 (0.00%)	1 (100.00%)	0 (0.00%)	0 (0.00%)	0 (0.00%)
ms4760-WH5	1	0 (0.00%)	1 (100.00%)	0 (0.00%)	0 (0.00%)	0 (0.00%)	0 (0.00%)
Total	140	3 (2.14%)	21 (15.00%)	34 (24.29%)	28 (20.00%)	24 (17.14%)	30 (21.43%)

Note: No. stands for number

## Discussion

In the present study, the African distribution of ms4760 *pfnhe1* polymorphisms from imported *P. falciparum* isolates in Wuhan during 2011–2016 was described. The number of DNNND repeats in Block II and NHNDNHNNDDD repeats in Block V ranged from 0 to 5 and 1 to 3, respectively. A total of 28 distinct ms4760 alleles were observed, including 23 referenced alleles [4, 33–35] and five that have not been previously characterized. A moderate level of *pfnhe1* microsatellite sequence polymorphisms was found (28 genotypes for 140 isolates). Previous studies observed more or less ms4760 microsatellite profiles of *pfnhe1*, including 8 ms4760 alleles in 71 *P. falciparum* isolates from Southeast Asia, Africa, and Latin America [20], 15 alleles in 88 isolates from western Kenya [31], and 27 alleles in 74 isolates from Congo [36].

Although no valid molecular marker of QNR is currently available, several studies have studied the connection between *pfnhe1* polymorphisms and QN susceptibility in vitro [20, 37–40]. Indeed, the QN sensitivities of isolates with different numbers of DNNND and NHNDHNNDDD repeats were compared [34]. Sequence polymorphisms of ms4760 in *pfnhe1* have been analyzed, especially in Block II and V [41]. Studies of the association of polymorphisms in Block II and V with in vitro susceptibility to QN have shown conflicting results. A study showed that more than one DNNND repeat in Block II and one NHNDNHNNDDD repeat in Block V in ms4760 were associated with reduced QN sensitivity in vivo [41]. The same study illustrated that isolates from Vietnam and the China-Myanmar border containing two or more DNNND repeats showed a much lower susceptibility to QN than those containing 0 or 1 repeats, and an increased number of NHNDNHNNDDD repeats was associated with high QN susceptibility in vitro [33, 42]. Further, a study supported that the IC_50_ of QN for parasites with 3 DNNND repeats was significantly higher than those with 1 or 2 repeats [31]. Thus, in the present study of parasite isolates from South Africa, parasites should be considered to have decreased susceptibility to QN because of the increasing trend in the number of DNNND repeats and the decreasing trend in the number of NHNDNHNNDDD repeats.

Several studies have demonstrated the different relationship between QN susceptibility in vitro and *pfnhe1* polymorphisms in isolates from western Kenya [31, 37]. These findings indicated that the presence of two DNNND repeats is linked to a decrease in QN susceptibility in vitro, and there was no association between the QN IC_50_ and NHNDNHNNDDD repeats [31, 37]. In one of the studies, parasites with one DNNND or NHNDNHNNDDD repeat were more susceptible to QN than those with more than one [37]. In addition, there was no association between QNR-associated Block II and Block V in *pfnhe1* in Thailand isolates [43] and Indian isolates [44]. Thus, QN susceptibility has been connected with polymorphisms in the *pfnhe1* ms4760 microsatellite in several studies [33, 42] but not in all.

These conflicting findings suggest that the role of *pfnhe1* ms4670 microsatellites in QNR may be dependent on the genetic background of the *P. falciparum* parasites [18] and/or their geographic origin [36]. Ms4760–7 with three DNNND repeats is currently found with high frequency among Asian parasites [20, 28] and shows significantly reduced sensitivity to QN compared to ms4760–1 (with two DNNND repeats) [45]. According to the variation in the increasing trend of DNNND repeats and the decreasing trend of NHNDNHNNDDD repeats from 2011 to 2016 in the current study, it can be proposed that imported *P. falciparum* isolates from Africa have reduced susceptibility to QN. Thus, attention should be paid to this issue.

Among the 140 studied sequences, West Africa displayed the highest number of different alleles, followed by South Africa, Central Africa, East Africa, and North Africa. While several alleles were shared between different countries, others appeared restricted to specific regions. Our genotyping data are consistent with previous findings, showing three profiles (ms4760–1, ms4760–3 and ms4760–7) as the predominant alleles. The newly observed profiles were from West Africa (4 isolates), Central Africa (2 isolates), East Africa (1 isolate) and South Africa (1 isolate), which suggests great abundance in the genetic diversity of *pfnhe1* in Africa. However, the sample sizes in this study were relatively small, particularly in 2011. Therefore, in vitro and in vivo studies with large-scale samples need to be considered for elucidating the role of *pfnhe1* ms4760 as a molecular marker of QNR.

## Conclusion

This primary study offers a comprehensive analysis of *pfnhe1* ms4760 polymorphisms from imported *P. falciparum* isolates in Wuhan. It demonstrated that parasite isolates from Africa are moderately diverse. In addition, continuous surveillance for molecular markers of QNR is highly recommended.

## Abbreviations

ACTs: Artemisinin based combination therapies
CDC: Centers for Disease Prevention and Control
DBSs: Dried-blood spots
gDNA: Genomic DNA.
*pfcrt*: *Plasmodium falciparum* chloroquine resistance transporter
*pfdhfr*: *Plasmodium falciparum* dihydrofolate reductase
*pfdhps*: *Plasmodium falciparum* dihydropteroate synthase
*pfK13*: *Plasmodium falciparum* kelch protein 13
*pfmdr1*: *Plasmodium falciparum* multidrug resistance 1
*pfmrp1*: *Plasmodium falciparum* multidrug resistance associated protein
*pfnhe1*: *Plasmodium falciparum* Na^+^/H^+^ exchanger
QN: Quinine
QNR: Quinine resistance
SNPs: Single nucleotide polymorphisms
WHO: World Health Organization
